# An evaluation of a *Stenotrophomonas maltophilia* outbreak due to commercial arterial blood gas collection kit

**DOI:** 10.1186/s13756-024-01410-8

**Published:** 2024-05-20

**Authors:** Esra Kazak, Uğur Önal, Nazmiye Ülkü Tüzemen, Funda Aslan, Gül Çalışkan, Hüsniye Şimşek, Zekiye Bakkaloğlu, Yasemin Numanoğlu Çevik, Yasemin Heper, Solmaz Çelebi, Emel Yılmaz, Mustafa Kemal Hacımustafaoğlu, Cüneyt Özakın, Emin Halis Akalın

**Affiliations:** 1 Department of Infectious Diseases and Clinical Microbiology, Faculty of Medicine, Bursa Uludag University, Gorukle, Nilufer, Bursa, Turkey; 2 Department of Medical Microbiology, Faculty of Medicine, Bursa Uludag University, Bursa, Turkey; 3 Infection Control Team, Faculty of Medicine, Bursa Uludag University, Bursa, Turkey; 4 Molecular Microbiology and Reference Laboratory, General Directorate of Public Health, Ministry of Health, Republic of Turkey, Ankara, Turkey; 5 Department of Pediatric Infectious Diseases, Faculty of Medicine, Bursa Uludag University, Bursa, Turkey

**Keywords:** Arterial blood gas collection kit, Hospital infections, Outbreak, Outbreak analysis, *Stenotrophomonas maltophilia*

## Abstract

**Background:**

*Stenotrophomonas maltophilia* is a gram-negative bacterium that can cause hospital infections and outbreaks within hospitals. This study aimed to evaluate an outbreak of *Stenotrophomonas maltophilia*, caused by ready-to-use commercial syringes containing liquid lithium and heparin for arterial blood gas collection in a university hospital.

**Methods:**

Upon detecting an increase in *Stenotrophomonas maltophilia* growth in blood cultures between 15.09.2021 and 19.11.2021, an outbreak analysis and a case-control study (52 patients for the case group, 56 patients for the control group) were performed considering risk factors for bacteremia. Samples from possible foci for bacteremia were also cultured. Growing bacteria were identified by matrix-assisted laser desorption ionization time-of-flight mass spectrometry. The genetic linkage and clonal relationship isolates were investigated with pulsed-field gel electrophoresis (PFGE) in the reference laboratory.

**Results:**

In the case-control study, the odds ratio for the central venous catheter [3.38 (95% confidence interval [CI]: 1.444, 8.705 ; *p* = 0.006)], for surgery [3.387 (95% confidence interval [CI]: 1.370, 8.373 ; *p* = 0.008)] and for arterial blood gas collection history [18.584 (95% confidence interval [CI]:4.086, 84.197; *p* < 0.001)] were identified as significant risk factors. *Stenotrophomonas maltophilia* growth was found in ready-to-use commercial syringes used for arterial blood gas collection. Molecular analysis showed that the growths in the samples taken from commercial syringes and the growths from blood cultures were the same. It was decided that the epidemic occurred because the method for sterilization of heparinized liquid preparations were not suitable. After discontinuing the use of the kits with this lot number, the outbreak was brought under control.

**Conclusions:**

According to our results, disposable or sterile medical equipment should be included as a risk factor in outbreak analyses. The method by which injectors containing liquids, such as heparin, are sterilized should be reviewed. Our study also revealed the importance of the cooperation of the infection control team with the microbiology laboratory.

## Background

*Stenotrophomonas maltophilia* is a gram-negative bacterium found in various environments, such as soil, plant roots, watery or moist surfaces, and plumbing systems [1]. Although it is accepted as a colonized or contaminant bacteria, it may cause lower respiratory tract and bloodstream infections. It can also be seen as a causative agent in various infections [2]. *S. maltophilia*, which is among the multi-drug resistant pathogens worldwide, can cause hospital infections and outbreaks. When examining hospital-acquired infections, they can be mortal, especially in immunocompromised patients or those who have undergone invasive procedures [3]. This study aims to evaluate an outbreak caused by *S. maltophilia* detected in hospitalized patients in a university hospital.

## Materials and methods

The observed hospital is a 900-bed tertiary hospital for adults and children with different clinics and eight intensive care units. In the hospital, consultations regarding infectious diseases are carried out by infectious disease and clinical microbiology specialists, and the infection control team nurses monitor the growth and infection control measures with active surveillance conducted throughout the hospital. During these consultations and during follow-ups, an increase in *S. maltophilia* growth in the clinical samples was noted. Therefore, growth was reviewed and detected in various cultures of 81 children and adult patients from different clinics between 15.09.2021 and 19.11.2021. Because this increase was significant when compared to all instances *S. maltophilia* growth in 2019 and 2020–2021, it was decided that an analysis be conducted.

First, patients with *S. maltophilia* growth between 15.09.2021 and 19.11.2021 in various clinical samples were examined. It was determined that blood culture growth was predominant among all types of growth. The case description was determined as ‘clinical and intensive care patients with at least one blood culture were positive for *S. maltophilia* between 15.09.2021–19.11.2021’. The outbreak analysis and case-control study were performed considering risk factors for bacteremia, such as catheter type, presence of IV solutions, drugs, blood and blood products. The control group included ‘patients with similar characteristics (age, clinic, etc.) but with no *S. maltophilia* growth in their blood culture over the same period’. In the comparison of *S. maltophilia* growth between the case and control groups, when the effect size was determined to be 20%, the minimum number of patients required to be included in the groups was determined to be 50 for 5% significance and 80% power.

The patients’ data were obtained from the hospital data processing system. The analysis included characteristics of samples with growth, growth dates, general characteristics of patients, prognosis, underlying diseases, clinics where the patients were hospitalized, whether the patients used antibiotics, surgery and haemodialysis, intubation history, whether interventional procedures were performed, bronchoscopy, enteral nutrition, history of total parenteral nutrition, blood product collection and history of potassium replacement use. Relevant procedure dates were also sourced from the patients.

### Epidemiological surveillance

Samples from possible foci (distilled water, sink water from patients’ rooms, heparinized flush solutions, treatment solutions [saline, dextrose, ringer lactate, etc.], alcohol, povidone-iodine, chlorhexidine and any syringes used on the patient) and unopened syringes within the same batch were cultured.

### Arterial catheter: blood gas sampling

Arterial blood gas tests were performed on patients with arterial catheters. Arterial catheters consisting of a standard arterial flush system were placed directly in the systemic arteries. The flush solution contained 2 U/mL heparin with 0.9% sodium chloride. Flush solution and arterial line connected with vascular three-way stopcock. First, the three-way stop cock was disinfected with 2% chlorhexidine, and then the nurse aspirated 2 mL of blood with sterile syringes. This blood sample was thrown into a medical waste container. Next, 1 mL of blood was aspirated with heparinized syringes using a three-way stopcock.

### Microbiologic identification and molecular analysis

Blood cultures were monitored using the BACTEC™ 9240 (Becton Dickinson, Sparks, MD, USA) fully automated blood culture device for 5 days or until bacterial growth was identified. Gram staining was prepared from the samples showing growth signals. The samples were inoculated on 5% sheep blood agar and eosin methylene blue (EMB) agar and incubated at 37 °C for 24–48 h. Matrix-assisted laser desorption ionization time-of-flight mass spectrometry (MALDI-TOF MS) (Bruker Daltonik, Bremen, Germany) was used to identify microorganisms reproduced in the media. The antibiotic susceptibility of these causative microorganisms was studied with the Phoenix™ 100 fully automated system and evaluated in accordance with the criteria of the European Committee on Antimicrobial Susceptibility Testing (EUCAST) [4].

The solutions, drugs and equipment thought to be a source for the outbreak (blood products, blood collection and component separation bags, liquid containing 0.9% NaCl, 5% dextrose, ready alcohol used in the fields, 2% chlorhexidine, hand antiseptic and alcohol prepared in the warehouse [70% diluted alcohol, tap water], the outer surface of multidose heparin vials taken from intensive care areas in which cases were common and heparin inside of the vials, and commercial kits consisting of syringes (containing liquid lithium and heparin used to collect arterial blood gas) were cultured. The samples were inoculated on 5% sheep blood agar and thioglycolate liquid and incubated at 37 °C for 24–48 h. MALDI-TOF was used to identify microorganisms with growth in the media.

On detecting the blood gas collection kit as a possible source of the outbreak, the outbreak analysis was repeated with this risk factor. After detecting the significant OR value in the second analysis, the blood culture samples and sterile blood gas collection kit samples were randomly selected and sent to a reference laboratory (National Antimicrobial Resistance Surveillance Laboratory) to investigate genetic linkage and clonal relationships. Bacterial identification of the 20 submitted strains (14 patients’ blood culture samples and 6 blood gas collection kits) was first confirmed by MALDI-TOF MS (Bruker Daltonics, Bremen, Germany). The source/epidemiological analysis of the 20 strains isolated from patients and medical equipment was performed with the PFGE method using the CHEF-DR III system (Bio-RadLaboratories-Belgium). In this test, bacterial cells were embedded in 1% low-melting-point agarose plugs (SeaKem® Gold Agarose, Lonza Rockland, USA) and lysed with lysozyme and proteinase K. The digestion of chromosomal DNA was carried out using SpeI (Thermo Scientific-Fermantas Corporation, Vilnius, Lithuania). DNA fragments were separated on 1% pulsed-field certified agarose (Lonza Rockland, USA) using a CHEF-DR III system (Bio-Rad Lab, Nazareth, Belgium) at 6 V/cm^2^ for 18 h at 14 °C and the pulse time was changed from 5 s to 70 s. Band profiles were analysed with BioNumerics version 7.5 software (Applied Maths, Sint-Martens-Latem, Belgium), and a similarity analysis was conducted using a Dice coefficient with a tolerance of 1.5% and an optimization of 1%. A dendrogram was constructed using the unweighted-pair group method with arithmetic mean (UPGMA). The clonal relationship among isolates was interpreted and evaluated according to the 1997 criteria developed by Tenover et al. [5]. Isolates with identical patterns were regarded as genotypically indistinguishable. Those with 1–3 band differences were considered closely related, those with 4–6 band differences were possibly related, and those with 7 or more band differences were considered unrelated or different.

### Patient follow-up

We conducted follow-ups for patients with potential colonizations or infections with contaminated arterial gas kits between September and December 2021. All inpatients who used heparinized syringes for blood gas measurement were monitored and followed to the last discharged patient. Furthermore, after the use of arterial blood gas collection kits was stopped, cultures with *S. maltophilia* growth were closely monitored for two months.

### Statistical methods

The results are presented as frequencies and percentages. Binary logistic regression was performed, and the crude odds ratios (OR) and their 95% confidence intervals (CIs) were used to test confounding variables associated with risk factors and reported. *p* < 0.05 was considered as the significance level. Statistical analyses were performed using IBM SPSS ver. 28.0 (IBM Corp. Released 2021. IBM SPSS Statistics for Windows, Version 28.0. Armonk, NY: IBM Corp.).

## Results

Examination of the 104 clinical samples with *S. maltophilia* growth between 15.09.2021 and 19.11.2021 in various clinical samples showed that most were seen between 1.10.2021 and 31.10.2021. Of those samples with growth, 72 (69%) were in the blood culture, 9 (8.6%) in the deep tracheal aspirate and 9 (8.6%) fluid samples were taken from the drain. Other samples are shown in Table 1.

**Table 1:** Distribution of samples with *Stenotrophomonas maltophilia* growth in their cultures between 15.09.-19.11.2021

Sample	Growth number (*n*)	%
Blood	72	69.2
EAS*	9	8.6
Sputum	7	6.7
Drain fluid	9	8.6
Peritoneal fluid	1	1
Bile	3	2.9
Pus	1	1
Conjunctiva	1	1
Urine	1	1

*EAS : Endotracheal aspirate samples

Based on the fact that 72 samples from 52 of the patients had *S. maltophilia* growth in the blood culture, all these patients fit the case description. The demographic and clinical characteristics of these patients and their underlying diseases are indicated in Table 2.

**Table 2:** The clinical characteristics of the blood culture positive patients for *Stenotrophomonas maltophilia*

	*n* (%)	
**Age** Children (≤ 18 years) Adult (> 18 years) (median year, average range)	16 (30.7%) 36 (69.2%) 39 (1 months − 87 years)	
**Gender (Female)**	30 (57%)	
**ICU**	32 (61.5%)	
**Baseline diseases**		
Hypertension Malignancy Leukemia Diabetes mellitus Hearth diseases Chronic renal failure Acute renal failure Liver disease COVID 19 Pulmonary disease Sepsis Acute gastroenteritis Intracranial abscess	9 (17%) 9 (17%) 3 (5.7%) 8 (15.3%) 13 (25%) 9 (17%) 2 (3.8%) 1 (1.9%) 10 (19.2%) 10 (19.2%) 2 (3.8%) 1 (1.9%) 1 (1.9%)	
**Catheter**	46 ( 88.4%)	
**Central venous catheter**	43 (83% )	
**Previous antibiotic usage**	40 (76.9%)	
Cephalosporins Carbapenem Piperacillin-tazobactam Sulbactam-ampisilin Aminoglycosides Glycopeptides	6 (11.5%) 30 (57.6%) 5 (9.6%) 2 (3.8%) 2 (3.8%) 16 (30.7%)	
**Bronchoscopy**	5 (9.6%)	
**Total parenteral nutrition**	10 (19.2%)	
**Operation**	21 (40.38%)	
**Interventional radiology**	2 (3.8%)	

Of the blood cultures, 45% were taken from the catheter and 55% from the peripheral veins. In the case-control study (52 patients in the case group and 56 patients in the control group), the odds ratio for the central venous catheter [3.38 (95% confidence interval [CI]: 1.444, 8.705 ; *p* = 0.006)], for surgery [3.387 (95% confidence interval [CI]: 1.370, 8.373 ; *p* = 0.008)] and for blood gas collection history [18.584 (95% confidence interval [CI]: 4.086, 84.197; *p* < 0.001)] were identified as significant risk factors, as shown in Table 3.

**Table 3:** Univariate logistic regression results of case-control study and associated risk factors among blood culture positive patients for *Stenotrophomonas maltophilia*

Variables	*Cases (*n* = 52)		**Control Group (*n* = 56)		*p*	OR	OR (95% CI)	
Age	16 (≤ 18 years) 36 (≥ 18 years)		18 (≤ 18 years) 38 (≥ 18 years)		0.500	0.996	0.983	1.008
Sex	28 (Female)		23 (Female)		0.247	1.572	0.730	3.386
Ward	32		30		0.824	1.091	0.506	2.353
	**Yes** **n(%)**	**No** **n(%)**	**Yes** **n(%)**	**No** **n(%)**				
CVC***	43 (83%)	9 (17%)	31 (55%)	25 (45%)	**0.006**	**3.545**	**1.444**	**8.705**
Whole blood transfusion	0 (0%)	52 (100%)	0 (0%)	56 (100%)	0.979	1.039	0.063	17.061
Erythrocyte transfusion	7 (13%)	45 (87%)	3 (5%)	53 (95%)	0.177	2.644	0.645	10.839
Thrombocyte transfusion	3 (6%)	49 (94%)	2 (4%)	54 (96%)	0.619	1.592	0.255	9.936
Fresh frozen plasma transfusion	3 (6%)	49 (94%)	5 (9%)	51 (91%)	0.500	0.600	0.136	2.649
Surgery	21 (40%)	31 (60%)	9 (16%)	47 (84%)	**0.008**	**3.387**	**1.370**	**8.373**
Interventional radiology	2 (4%)	50 (96%)	7 (13%)	49 (87%)	0.112	0.269	0.053	1.359
Blood gas collection history	50 (96%)	2 (4%)	31 (55%)	25 (45%)	**< 0.001**	**18.548**	**4.086**	**84.197**

*Patients with *Stenotrophomonas maltophilia* bacteremia

** Patients without *Stenotrophomonas maltophilia* bacteremia

*** CVC: Central venous catheter

Among the cultured medical instruments that were thought to be a source for the break, such as liquids containing 0.9% NaCl and 5% dextrose, sample taken from tap water, the alcohol sample, heparin liquid, swab sample taken from the outer surface seal of the heparin vial, none of the samples showed *S. maltophilia* growth.

Microbiological analysis was performed from 11 randomly selected, ready-to-use commercial syringes containing liquid lithium and heparin for arterial blood gas collection. The 24-, 48- and 72-hour evaluations of cultures showed growth; *S. maltophilia* was found in 6 samples cultured in solid and liquid media. These microbiological findings were consistent with case control study results, as shown in Table 3.

**Fig. 1:**
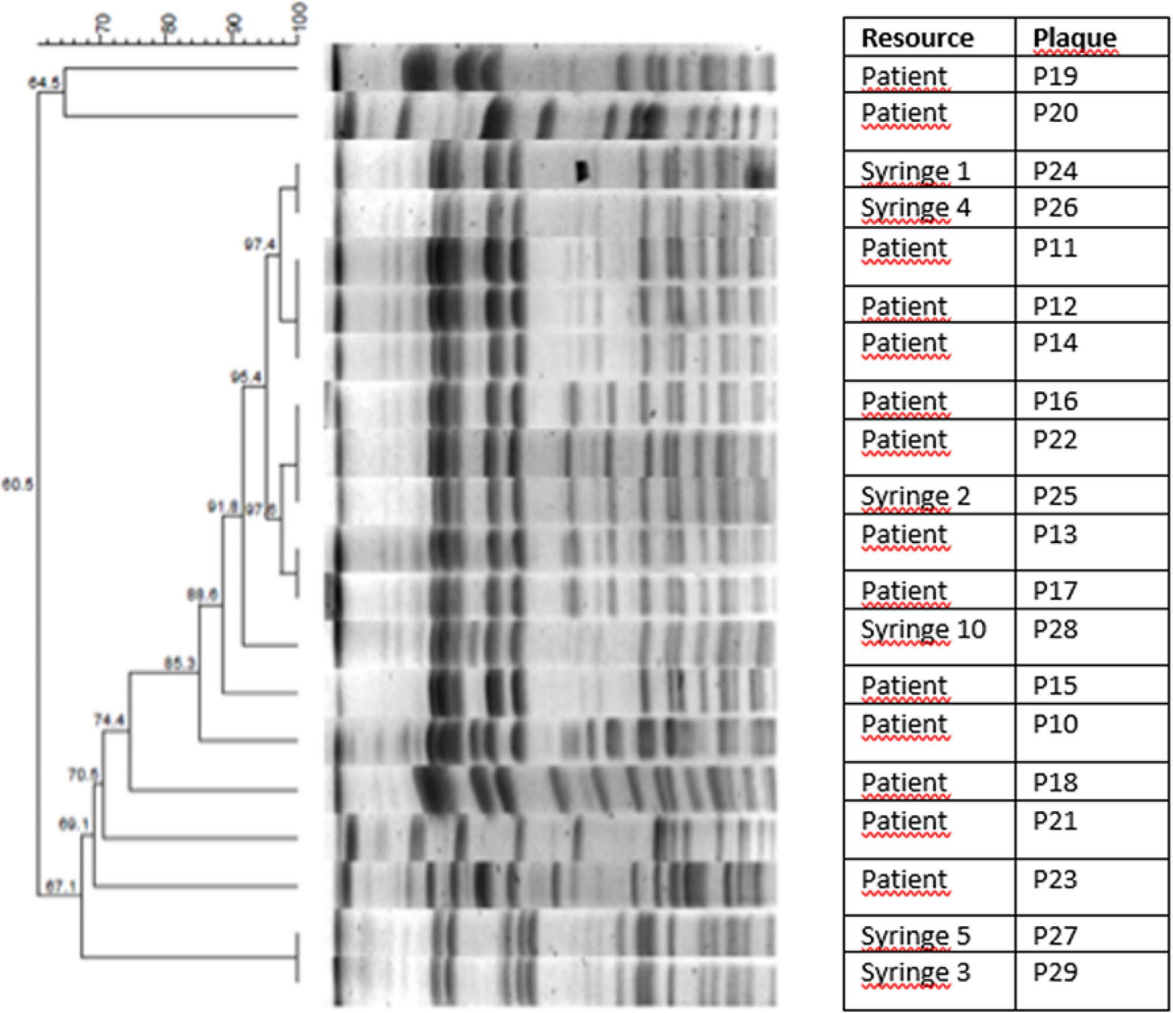
The dendrogram showing the clonal relationship of the strains

The clonal relationships of 20 randomly selected isolates (14 patients’ blood culture samples and 6 sterile blood gas collection kits) were investigated with PFGE in the reference laboratory. 7 Different PFGE groups were identified, of which only 5 contained 1 strain. The other two PFGE groups contained cluster-forming strains, and the cluster width varied between 2 and 13. The clustering rate was determined to be 65%. Considering the similarity rate of ≥ 85% among PFGE profiles, 5 out of 20 strains showed specific profiles, while the remaining 15 strains were classified into 2 different epidemiologically related groups. A clonal relationship was found between isolates with plate numbers 24, 25, 26 and 28 (injector) and plate numbers 11, 12, 13, 14, 16, 17, 19, 20 and 22 (patients) included in the cluster of 13 strains. The isolates with plate numbers 27 and 29 (injectors) were found to be the same but were not associated with other isolates. The isolates with plate numbers 10, 15, 18, 21 and 23 (belonging to patients) were considered specific profiles and were not associated with any isolate. The dendrogram that shows the clonal relationships of the strains is shown in Fig. 1. A clonal association was determined between the isolates of commercial blood gas collection kits and blood culture samples.

Based on these results, the ready-to-use blood gas collection kits, which were determined as the source, were discontinued. These syringes were withdrawn from all units and returned to the manufacturer. The infection control team trained the entire hospital staff and explained the rules to be followed regarding blood gas sampling.

**Fig. 2:**
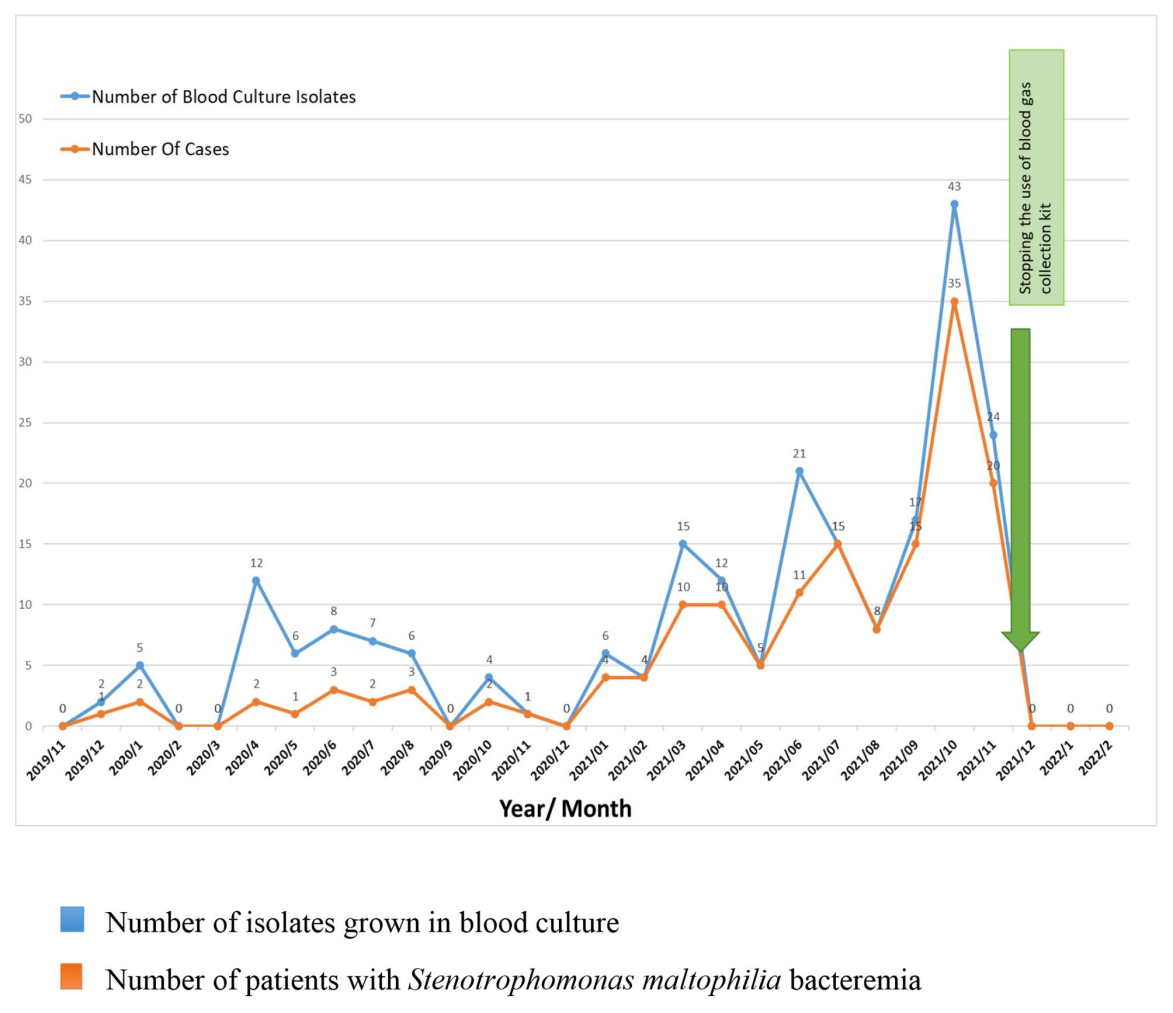
The outbreak curve of *Stenotrophomonas maltophilia*

A report was immediately sent to the Ministry of Health on December 2021. After that, the Ministry of Health sent a definitive letter to all hospitals to stop using these commercial blood gas collection kits. In the following days, patients with *S. maltophilia* growth in their blood cultures were followed. It was observed that growth stopped in the blood culture after the kits were discontinued. The epidemic curve, which includes the distribution of *S. maltophilia* isolates grown in blood culture and the number of patients with *Stenotrophomonas maltophilia* bacteremia by months, is shown in Fig. 2. This significant regression in growth is also shown by the outbreak curve in this figure.

The outbreak was controlled by stopping the usage and sending back the heparinized blood gas syringes belonging to the same manufacturing batch with the same serial code.

## Discussion

*S. maltophilia* are mostly found in aquatic environments and soil. Bacteria that are generally considered contaminant bacteria can be found on environmental surfaces and medical instruments within the hospital [6]. As an agent that can settle on many surfaces, including sterile areas, such as catheters and prostheses, and form a biofilm, *S. maltophilia* can cause disease in patients who have undergone invasive procedures and or have compromised immune systems. Bacteremia, which often affects the respiratory tract, is common in patients hospitalized for a long time who use broad-spectrum antibiotics such as carbapenem or are immunosuppressed. This can lead to endocarditis and infections of the central nervous system and urinary system [7]. The resistant bacteria can also cause outbreaks in hospitals [8, 9]. Long-term hospitalization and a history of antibiotic use, as well as invasive procedures such as mechanical ventilation, were determined to be the most important risk factors for the development of infection [9].

Although this bacterium is generally reported to be of low virulence, *S. maltophilia* bacteremia may be fatal in some patients [10]. Between 2002 and 2016, 140 patients with nosocomial *Stenotrophomonas maltophilia* bacteremia were examined in a university hospital, and the mortality rate on the 14th day was reported to be 32.9%. It has been shown that starting antibiotics as soon as possible and withdrawing the central venous catheter is essential for patients’ survival [11].

Outbreaks with this bacterium caused by disinfectants or different sterile solutions have been reported for a very long time [12]. When examining outbreaks due to *S. maltophilia* reported in the literature, it was found that water and medical equipment were identified as possible sources in the foreground. However, outbreaks can be seen in different forms, such as in the contamination of dialysates used in dialysis centres or acute endophthalmitis outbreaks detected in eye centers where intravitreal injections are applied [13–15]. A study conducted in Mexico examined the blood and urine cultures of pediatric patients in the emergency room together with 21 *S. maltophilia* isolates taken from the taps of the same unit and found a total of 7 PFGE clones with 52% genetic variation [16]. Similarly, 7 different PFGE groups were identified in the current study. Only 5 of them contained 1 strain, and the similarity rate between PFGE profiles was considered as ≥ 85%. While 5 out of 20 strains showed specific profiles, the remaining 15 strains were classified into 2 different epidemiologically related groups. The use of multiple-dose vials can also be a source of these bacteria. In fact, as a result of the use of a vial containing contaminated bevacizumab solution in different patients in India, acute endophthalmitis developed in 28 patients within 3 days [17]. However, contaminated, ready-made and disposable preparations can also cause outbreaks. It was reported in the USA that between 2004 and 2005, 36 patients from different states developed infections due to a heparin-saline flush solution that was contaminated with *Pseudomonas fluorescens* during production [18]. Dias et al. evaluated 154 patients in their hospital who they thought had been given a ready-made heparin catheter lock solution thought to be contaminated, 32 individuals (out of 48 who had a central venous catheter) had *Pseudomonas putida* growth in their blood cultures, and 9 patients had *S. maltophilia* growth in their blood cultures. A molecular analysis also revealed that growth in solutions thought to be contaminants was similar to that of *S. maltophilia* growth in blood cultures [19].

Our study began with an observation of the increased blood culture growth over a two month period. On the genetic similarity of the growths in the samples taken from the disposable, ready-to-use blood gas collection kit with the growths in the patient blood cultures, the use of kits with this lot number was stopped, and the epidemic was brought under control. We think that the heparinized syringe and the three-way stop cock come into contact for a while, and possible contact contamination occurs. Afterwards, in order to keep the arterial tract open, 5 ml of heparinized solution was drawn with a new and clean syringe and given back to the arterial line. In possible contamination of the three-way stop cock, this contaminant was transferred to the arterial line. Menekse et al. [20] reported a commercial needle blood gas injector as the source of a *S. maltophilia* outbreak over the same period as the present study. They also reported that their investigation for blood gas kits started after the letter from the Ministry of Health was released.

The aseptic manufacturing process is essential to producing sterile products and minimising the contamination risk for patients. It involves multiple steps under sterile conditions for the handling of materials and medical equipment [21]. Gas sterilization with ethylene oxide is still a common method used for sterilizing some medical devices; however, it was found that this method is not sufficient for the sterilization of liquid products due to limitations of penetration depth [22]. Similarly, gamma irradiation is used for various disposable product sterilizations [23]. However, this method bears the risk of changing the composition of the heparin. Other methods should be investigated for heparinized solutions.

### Limitations of the study

In our study, all strains of *S. maltophilia* isolated from blood cultures were not subject to molecular investigation. However, a link between the possible source and blood culture isolates was demonstrated in the samples studied.

## Conclusions

Disposable or sterile medical equipment should be included in outbreak analyses as a potential source for such outbreaks, especially for bacteria that can be found in liquid media. The appropriate methods for sterilization of such heparinized liquid preparations are still unclear and this issue should be discussed. This study also shows the importance of the work of infection control teams and their cooperation with microbiology laboratories, the review and identification of resources, including disposable, ready-made preparations, and taking appropriate precautions in preventing nosocomial infections.

## Data Availability

No datasets were generated or analysed during the current study.

## Abbreviations

EUCAST: European Committee on Antimicrobial Susceptibility Testing
MALDI-TOF MS: Matrix-assisted laser desorption ionization time-of-flight mass spectrometry
PFGE: Pulsed-field gel electrophoresis
UPGMA: Unweighted-pair group method with arithmetic mean
